# A FRET method for investigating dimer/monomer status and conformation of the UVR8 photoreceptor†
†Electronic supplementary information (ESI) available. See DOI: 10.1039/c8pp00489g


**DOI:** 10.1039/c8pp00489g

**Published:** 2018-12-04

**Authors:** Xinyang Liao, Ben Zhang, Michael R. Blatt, Gareth I. Jenkins

**Affiliations:** a Institute of Molecular , Cell and Systems Biology , College of Medical , Veterinary and Life Sciences , Bower Building , University of Glasgow , Glasgow G12 8QQ , UK . Email: Gareth.Jenkins@Glasgow.ac.uk

## Abstract

The photoreceptor UVR8 has a pivotal role in mediating plant responses to UV-B wavelengths. Dimeric UVR8 dissociates into monomers following UV-B photoreception, and there is evidence that this process is accompanied by conformational changes that may facilitate interaction of UVR8 with other proteins to initiate signaling. Hence monitoring UVR8 dimer/monomer status and conformation is key to understanding UVR8 action. Here we have used Fluorescence Resonance Energy Transfer (FRET) to study these processes in both wild-type and mutant UVR8 proteins *in vivo*. UVR8 was fused to GFP and mCherry at the C- and N-termini, respectively and both the FRET efficiency and loss of GFP fluorescence after photobleaching were measured. In addition, measurements were made for UVR8 fused to either GFP or mCherry to eliminate intra-molecular FRET signals. The results indicate that dissociation of UVR8 dimer to monomer principally accounts for the loss of FRET signal for wild-type UVR8 and there is little evidence of a contribution from conformational change *in vivo*. Examination of plants expressing UVR8^W285F^ and UVR8^D96N,D107N^ are consistent with these mutant proteins being constitutively dimeric and monomeric, respectively. The methods employed here will be valuable for monitoring UVR8 dimer/monomer status *in vivo* in relation to signaling, and will facilitate characterization of dimer/monomer status and conformation of further UVR8 mutants.

## Abstract 2

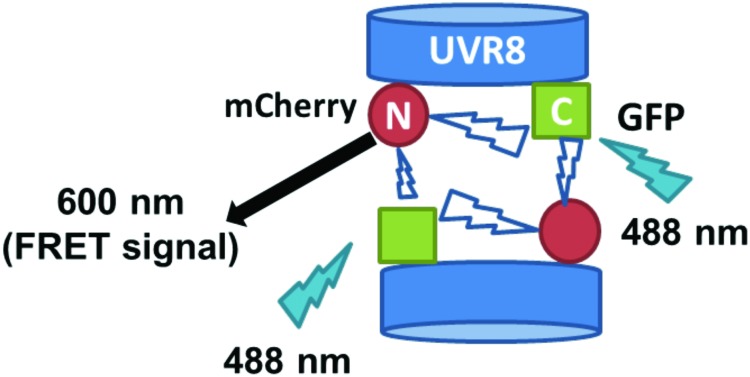
A Fluorescence Resonance Energy Transfer (FRET) method is used to monitor dimer/monomer status and conformation of both wild-type and mutant variants of the UV-B photoreceptor UVR8 *in vivo*.

## Introduction

Plants exhibit numerous responses to UV-B wavelengths (280–315 nm), many of which are likely to be mediated by the UV-B photoreceptor UV RESISTANCE LOCUS 8 (UVR8). Studies of *Arabidopsis thaliana* mutants lacking UVR8 show that the photoreceptor initiates a range of biochemical, morphogenic and physiological responses underpinned by extensive, UV-B induced changes in gene expression.^1^,^2^

UVR8 is a 7-bladed β-propeller protein that exists as a homodimer in the absence of UV-B.^3^,^4^ UV-B exposure induces dissociation of the dimer to produce monomers that initiate downstream responses through interaction with the protein CONSTITUTIVELY PHOTOMORPHOGENIC 1 (COP1)^5^ and with specific transcription factors.^6^,^7^ Further proteins, termed REPRESSOR OF UV PHOTOMORPHOGENESIS (RUP) proteins, interact with UVR8 to displace COP1 and promote re-association of monomers to form the dimer.^8^ Under photoperiodic illumination with light containing UV-B both monomer formation and re-dimerization occur, resulting in establishment of a photo-equilibrium of UVR8 dimer and monomer.^9^

There is evidence that UV-B photoreception by UVR8, with the resulting monomerization results in conformational changes to the protein.^5^,^10^–^13^ However, since the N- and C-termini do not appear in the crystal structures of UVR8,^3^,^4^,^12^ detailed information about their location and conformational changes following monomerization are lacking. It is proposed that conformational changes are likely to increase accessibility of the C-terminus of monomeric UVR8 to COP1. The interaction between UVR8 and COP1 is UV-B-dependent^14^ and involves binding to a specific region within the C-terminus.^15^,^16^ A recent study^13^ indicates that the UVR8 monomer can adopt multiple conformations with an extended C-terminus.

Fluorescence Resonance Energy Transfer (FRET) is a commonly used method in confocal microscopy to monitor interactions between two proteins. It involves the use of two different fluorescent molecules, such that excitation of one results in radiationless excitation energy transfer that activates the other, leading to a fluorescent signal that can be measured. FRET will only occur if the donor and acceptor molecules are in sufficiently close proximity. For example, in plant photobiology FRET has been used to show an interaction between phytochrome B and cryptochrome 2 photoreceptors.^17^ However, FRET can also be adapted to monitor the proximity of two regions within the same molecule; such intramolecular or ‘single-molecule’ FRET involves introducing two fluorophores into the molecule, often at the N- and C-termini. For example, this approach has been used elegantly to study the structural dynamics of potassium channels^18^,^19^ and to monitor the voltage-driven conformational changes associated with channel voltage-sensor domain interactions with vesicle trafficking proteins.^20^

FRET has the potential to provide valuable insights into UVR8 molecular function. The extent of monomer formation is key to UVR8 function, because the monomer is the form that initiates signaling. As reported here, FRET enables the dimer/monomer status of UVR8 to be monitored *in vivo*, both in relation to environmental stimuli and also in plants expressing specific mutant UVR8 proteins. Various mutations have been found to alter the strength of the UVR8 dimer, and in some cases constitutively dimeric and monomeric mutant proteins have been identified.^3^–^5^,^21^,^22^ Moreover, intra-molecular FRET has the potential to monitor conformational changes in the UVR8 protein, either as a result of UV-B exposure or in mutant proteins. In this study we developed FRET-based methods for monitoring the relative positions of the N- and C-termini of UVR8 to gain insights into UVR8 dimer/monomer status and conformation. In addition, the methods employed here will facilitate characterization of further UVR8 mutants *in vivo*.

## Materials and methods

### Preparation of the vectors for FRET experiments

Gateway® Technology (Thermo Fisher Scientific) was used to create constructs used for the FRET experiments. The pDONR207 vector was used as the donor vector for the pFRET-NcCg-DEST vector. The pDONR221-P1P4 vector and pDONR221-P3P2 vector were used as the donor vectors for pFRET-2in1-DEST vector.^19^,^20^,^23^ A PCR product of the target gene fused with the B recombination sites was transferred into the donor vector by the BP reaction using Gateway™ BP Clonase™ II Enzyme mix (Thermo Fisher Scientific) following the manufacturer's instructions. Then, the BP product was transformed in *E. coli* to select positive vector. The expected donor vector was transferred into the destination vector by the LR reaction using Gateway™ LR Clonase™ II Enzyme mix (Thermo Fisher Scientific) following the manufacturer's instructions. The product destination vectors were checked by restriction enzyme digestion and sequencing. Finally, the verified destination vectors were transformed in *Agrobacterium* GV3101 for subsequent transient expression in tobacco. The primers used in all steps are listed in Table S1.†

### Transient transformation in *Nicotiana tabacum* leaf

A single colony of *Agrobacterium* containing the required construct was picked into 10 ml of liquid LB medium containing appropriate antibiotics. The culture was incubated at 28 °C with 200 rpm shaking overnight. The following day, the OD_600_ values of the culture were measured and when it reached around 0.6–1.0 cells were spun down at 4000*g* for 5 min and the supernatant was discarded. The pellets were washed with distilled water twice. Then, cells were resuspended in an appropriate volume of the infiltration buffer (10 mM MgCl_2_, 10 mM MES, 100 μM acetosyringone, pH 6.5), making its OD_600_ reach 0.3–0.4. This *Agrobacterium* suspension was used for infiltration after 3 hours incubation at 4 °C.

Four to six-week-old *Nicotiana tabacum* plants were used for leaf infiltration for transient transformation. Plants were well watered on the day before infiltration. The lower epidermis of the chosen leaf was grazed by a blade or a needle. Then, the prepared *Agrobacterium* suspension was injected into the wound on the abaxial leaf epidermis by a needle-less 1 ml syringe. Plants were moved back to the growth chamber and the expression was observed after 2–3 days by confocal microscopy.

**Fig. 1:**
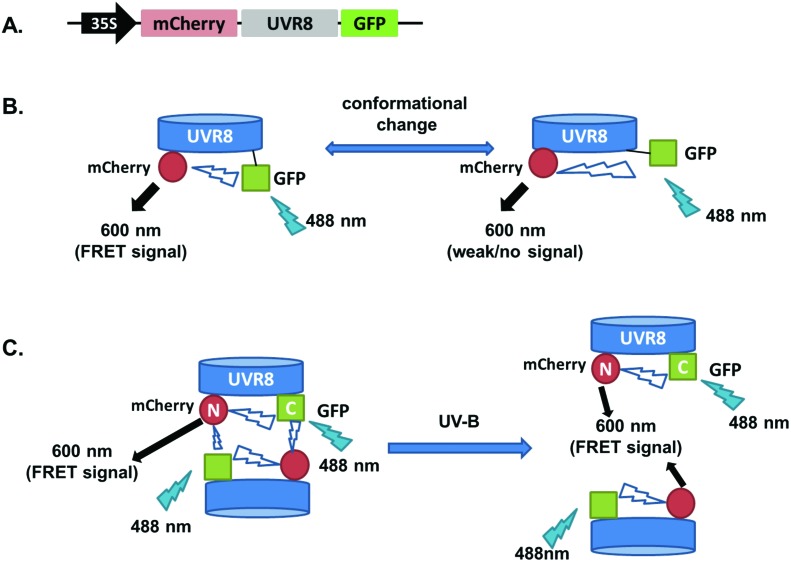
Schematic diagram showing FRET with the UVR8 construct inserted into the pFRET-NcCg-DEST vector. A. Sketch of the UVR8 construct in the pFRET-NcCg-DEST vector. B. The working principle of the UVR8 construct. GFP is excited by 488 nm light and excites mCherry, causing fluorescence at 600 nm. This mCherry fluorescence signal is collected as the FRET signal. If there is a conformational change of UVR8 that affects the relative positions of the N- and C-termini, the distance between GFP and mCherry will change, so the FRET signal will be reduced or enhanced. C. Origin of the UVR8 FRET signal with pFRET-NcCg-DEST vector. For UVR8 dimer, the FRET signal potentially comes from two sources, the intramolecular signal of the monomer and inter-monomer signal in the dimer. After UV-B induced monomerization, the homodimer is dissociated and only the intramolecular FRET signal remains.

**Fig. 2:**
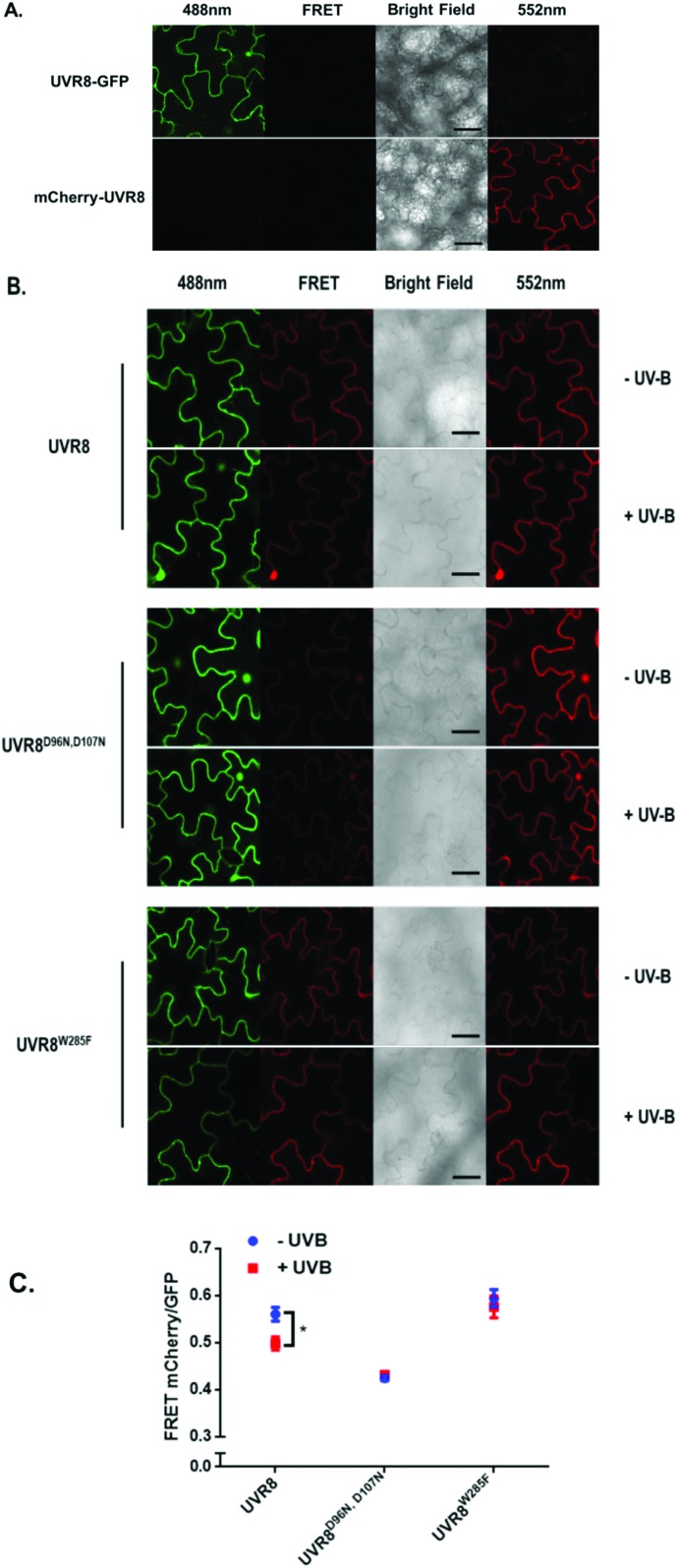
FRET results obtained with the pFRET-NcCg-DEST vector. A. Images were captured from tobacco leaves, not exposed to UV-B, transiently expressing the negative controls, UVR8-GFP and mCherry-UVR8 from the pFRET-NcCg-DEST vector. Images from left to right are GFP fluorescence signal excited with 488 nm light, mCherry fluorescence signal excited with 488 nm light (FRET), bright field, and mCherry fluorescence signal excited with 552 nm light. Bar = 50 μm. B. Images were captured from tobacco leaves transiently expressing mCherry-UVR8-GFP, mCherry-UVR8^D96N,D107N^-GFP and mCherry-UVR8^W285F^-GFP from the pFRET-NcCg-DEST vector (fusions abbreviated in the figure panels to UVR8, UVR8^D96N,D107N^ and UVR8^W285F^). Images from left to right as in A. Plants were exposed (+UV-B) or not (–UV-B) to 3 μmol m^–2^ s^–1^ broadband UV-B for 1 hour before taking images. C. Means ± S.E. of FRET ratios from 5 independent experiments of constructs in B. For each experiment, data were collected from 10 images and 20 regions of each image were selected at random to calculate the mean fluorescence intensity. The FRET efficiency was calculated as the mean fluorescence intensity ratio [mCherry (488)/GFP (488)]. *Indicates significance at *P* < 0.05.

Where indicated, leaves were exposed to broadband UVB-313 fluorescent tubes (Q-Panel Co., USA; Fig. 2-1C) covered by cellulose acetate film (FLM400110/2925, West Design Products) to filter out UV-C. The spectrum of the UV-B source is published in Cloix *et al.* (2012). A Skye Spectrosense 1 meter (Skye Instruments) with a SKU 430 sensor (Skye Instruments) was used to measure the UV-B fluence rates.

### Confocal microscopy

FRET studies were carried out as described by Zhang *et al.* (2017).^19^ GFP and mCherry fluorescence were observed by a Leica TCP SP8 FRET-FLIM confocal microscope (Leica TCP SP8) with 65 mW argon lasers and a 20×/0.75 NA objective lens. For the FRET signal, GFP fluorescence was excited by the 8% 488 nm laser and collected over 500 to 535 nm with 30% gain value. Meanwhile, mCherry fluorescence was collected over 590 to 645 nm with 100% gain value. mCherry also was separately excited by the 8% 552 nm laser as a mCherry control. The FRET efficiency was calculated as described in the figure legends. For the photobleaching experiments, five scans were taken with the 8% 488 nm laser to provide the pre-bleach data, before photobleaching for 50 seconds with 100% 552 nm light followed by five scans with the 8% 488 nm laser to obtain the post-bleach data. The FRET efficiency was calculated as described in the figure legends.

### Statistical analysis

Statistical analysis of the data was performed using two-way ANOVA with the Šídák *post-hoc* test on GraphPad prism software.

## Results

### Measurements of FRET efficiency

The FRET-pair tags Green Fluorescent Protein (GFP) and m-Cherry were fused to UVR8 protein in the pFRET-NcCg-DEST vector.^19^ As shown in Fig. 1A, the FRET donor GFP is fused at the C-terminus of UVR8 and the acceptor mCherry is fused at the N-terminus. When GFP is excited using a 488 nm laser, the mCherry could be excited by energy transfer from GFP. A change in the conformation of UVR8 (Fig. 1B), or conversion from dimer to monomer (Fig. 1C) may alter the distance between mCherry and GFP, leading to a change in FRET efficiency. Furthermore, since UVR8 can exist as either a homodimer or monomer, fusion constructs with the constitutively monomeric mutant UVR8^D96N,D107N^ (ref. 22) and the constitutively dimeric mutant UVR8^W285F^ (ref. 3–5 and 21) were created to assess the impact of dimer/monomer status on FRET efficiency. Additionally, UVR8 fusions with only GFP or only mCherry were generated as negative controls. All constructs were transiently expressed in 4- to 6-week-old tobacco leaves. Protein expression and dimer/monomer status were checked 3 days after infiltration by western immunodetection with the appropriate antibodies (Fig. S1†).

As shown in Fig. 2A, the expected fluorescence is seen when the negative control proteins UVR8-GFP and mCherry-UVR8 are separately excited at 488 nm and 552 nm respectively, but no FRET signal was seen following excitation at 488 nm, since this would require the presence of UVR8 fused to both GFP and mCherry. FRET images for the UVR8 FRET-pair constructs were captured under the same microscopy settings (Fig. 2B) and the FRET efficiency of these constructs was quantified. The FRET efficiency was calculated as the ratio of mean fluorescence intensity between FRET signal [mCherry (488)/GFP (488)] and GFP signal in randomly selected areas (Fig. 2C). The data for wild-type UVR8 show a relatively high FRET signal prior to UV-B exposure, when the protein is in the dimeric form, and a decrease in FRET efficiency following UV-B exposure, when dimers dissociate into monomers. For the UVR8 dimer, the FRET signal could potentially come from two components (Fig. 1C): (i) energy transfer from the C-terminus to the N-terminus of the same monomer molecule; (ii) energy transfer between the two monomer molecules in the dimer. When UVR8 is monomerized by UV-B, or because of mutations, the second source of the FRET signal is lost, but the first type of FRET signal is still possible.

Examination of the UVR8 mutant fusions shows a correlation between FRET efficiency and dimer/monomer status (Fig. 2C). The FRET value of the constitutively dimeric UVR8^W285F^ is relatively high whereas that for constitutively monomeric UVR8^D96N,D107N^ is relatively low. In both mutants the FRET efficiency is unaffected by UV-B treatment. Moreover, results for UVR8^D96N,D107N^ demonstrate that the distance between the N-terminus and C-terminus of UVR8 is close enough for FRET, indicating that this method is suitable to investigate any conformational change of UVR8. However, there is no evidence for any conformational change for UVR8^D96N,D107N^ following UV-B exposure. Since there is a large change in FRET efficiency between the dimeric and monomeric mutants, it is likely that the decrease in FRET signal in wild-type UVR8 following UV-B exposure is principally due to dissociation of the dimer to produce monomers.

### Measurement of FRET by photobleaching

**Fig. 3:**
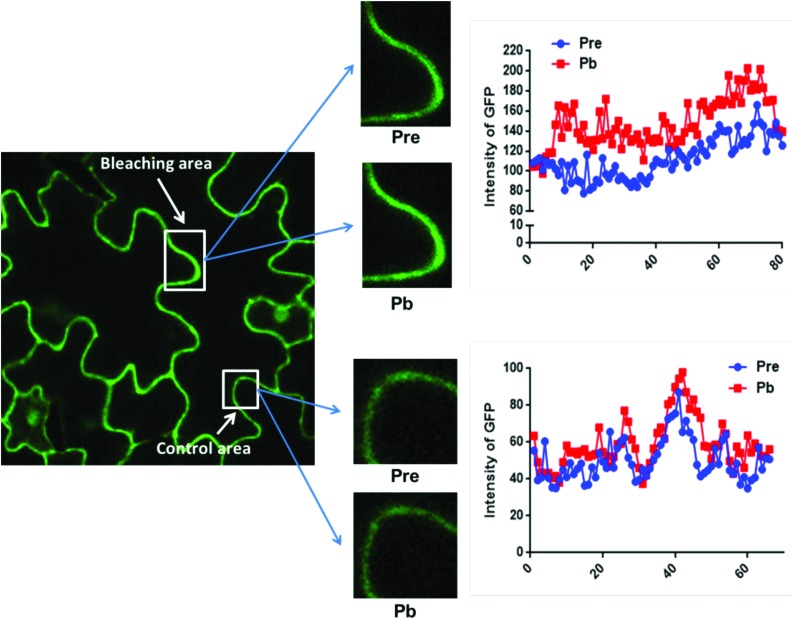
The method of photobleaching. For each selected area, images are captured before and after photobleaching (bleaching area) with 552 nm light for 50 seconds. Another area without photobleaching is selected as the control area. GFP was excited with 488 nm light. GFP fluorescence intensity before (Pre) and after (Pb) photobleaching at 100 points along the periphery of the cell in both selected areas is shown.

**Fig. 4:**
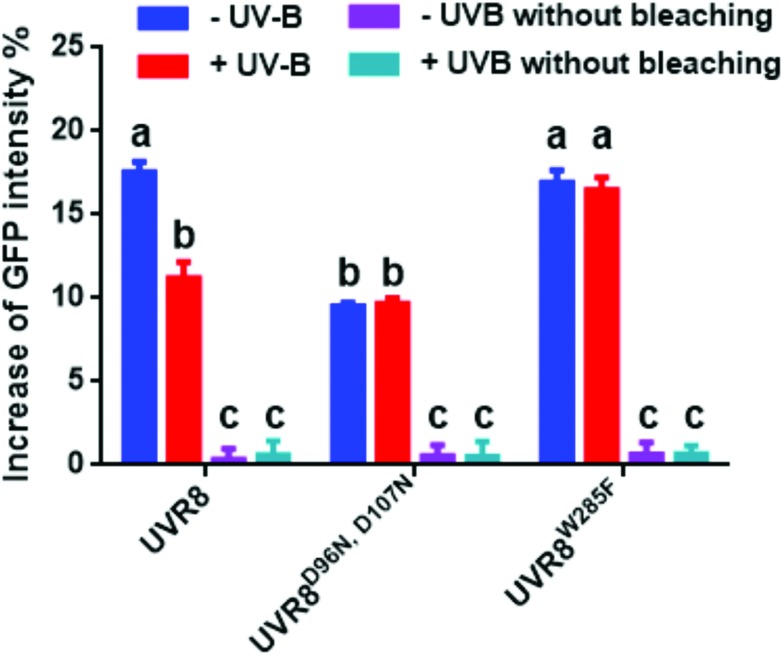
Photobleaching results with the pFRET-NcCg-DEST vector. GFP fluorescence intensity was analysed before and after photobleaching for mCherry-UVR8-GFP, mCherry-UVR8^D96N,D107N^-GFP and mCherry-UVR8^W285F^-GFP expressed from the pFRET-NcCg-DEST vector (fusions abbreviated in the figure to UVR8, UVR8^D96N,D107N^ and UVR8^W285F^). Plants were exposed (+UV-B) or not (–UV-B) to 3 μmol m^–2^ s^–1^ broadband UV-B for 1 hour before being used for experiments. Primary data obtained as described in Fig. 3 are shown in Fig. S3,† together with the negative control results. The percentage increase in GFP fluorescence intensity after photobleaching for each construct was calculated by [(*I*_Pb_ – *I*_Pre_)/*I*_Pre_] × 100, where *I*_Pre_ and *I*_Pb_ indicate GFP intensities in the bleaching area before and after photobleaching, respectively. Means with S.E. are shown (*n* > 5). Significance at *P* < 0.01 is indicated by letters.

The data in Fig. 2C were obtained by calculating the intensity ratio of mCherry and GFP signals and this method is based on the sensitized emission of both donor and acceptor. However, there are several potential sources for error, such as background interference, spectral bleed-through and the protein expression level. Considering these limitations and to verify previous results, another method was applied. As described previously, the principle of FRET is that the acceptor is excited by radiationless energy transfer from the donor. During this process, the donor loses part of its energy due to the energy resonance effect. Therefore, measuring the decrease of the donor (GFP) energy caused by FRET is another approach to test the FRET efficiency. Fig. 3 presents the steps of this method. For each sample, first the GFP fluorescence image was taken under the 488 nm laser. Next, a randomly selected area was bleached for 50 seconds with 100% 552 nm laser, the excitation wavelength of mCherry. Then, another image of GFP was captured. The strong excitation of mCherry eliminates FRET from GFP and consequently increases the GFP fluorescence; the increase in GFP fluorescence after photobleaching therefore indicates the extent of FRET to mCherry. The GFP intensity was measured at approximately 100 points along the periphery of the cell in the selected area. The blue (Pre) and red (Pb) lines in Fig. S3A† represent the intensity of GFP before and after photobleaching, respectively, and these values are plotted against the individual points along the periphery of the cell in the selected area (as shown by the number along the *x* axis in Fig. S3A†). Meanwhile, another area without photobleaching was analysed by the same method as a negative control (Fig. S3B†). The percentage increase in GFP fluorescence intensity following photobleaching (Fig. 4) was calculated as [(*I*_Pb_ – *I*_Pre_)/*I*_Pre_] × 100, where *I*_Pre_ and *I*_Pb_ indicate the mean GFP intensity in the bleaching area before and after photobleaching, respectively.

The primary photobleaching data for UVR8, UVR8^D96N,D107N^ and UVR8^W285F^ are shown in Fig. S3A† and the corresponding negative controls are shown in Fig. S3B.† For the negative controls, there is no significant difference of GFP intensity between pre-bleaching and post-bleaching for all samples. For wild-type UVR8, the percentage increase in GFP fluorescence after photobleaching is greater before than after UV-B exposure (Fig. 4), indicating that UV-B causes a decrease in FRET. The photobleaching of UVR8^W285F^ leads to a strong increase of GFP fluorescence intensity, similar to that for wild-type UVR8 without UV-B, whereas UVR8^D96N,D107N^ shows a smaller change, similar to wild-type UVR8 after UV-B treatment. The fluorescence signals for both mutants are unaffected by UV-B treatment (Fig. 4). These results are consistent with the FRET efficiency data (Fig. 2C) and indicate that FRET is greater in dimeric UVR8 than in the monomeric proteins.

### Measurement of FRET using the pFRET-2in1-DEST vector

**Fig. 5:**
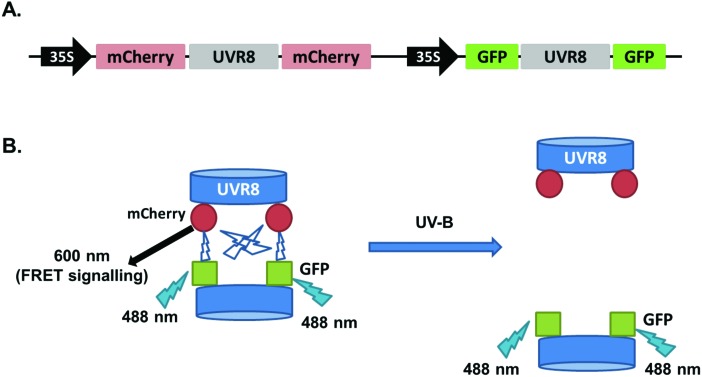
Schematic diagram of the construct of UVR8 made in the pFRET-2in1-DEST vector. A. Sketch of the construct in the pFRET-2in1-DEST vector used to transiently express mCherry-UVR8-mCherry and GFP-UVR8-GFP proteins in tobacco. B. Illustration describing the working principle of the UVR8 construct. Excitation of GFP on one monomer in the dimer can excite mCherry on the other monomer, causing fluorescence at 600 nm (the FRET signal). Monomerization will increase the distance between GFP and mCherry, so the FRET signal will be lost.

In the above experiments both intermolecular FRET and intramolecular FRET can contribute to the final FRET signal and these two sources may interfere with each other. To reduce the complexity of the experimental system, new constructs were generated in the pFRET-2in1-DEST vector,^23^ which allows expression of two individual protein fusions with different tags in a 1 : 1 ratio at the same time. We produced a vector to express mCherry-UVR8-mCherry and GFP-UVR8-GFP proteins together. As shown in Fig. 5, UVR8 dimer should have a strong FRET signal due to the intermolecular FRET but in monomeric UVR8 this signal should be removed and there should be no contribution from intramolecular FRET.

**Fig. 6:**
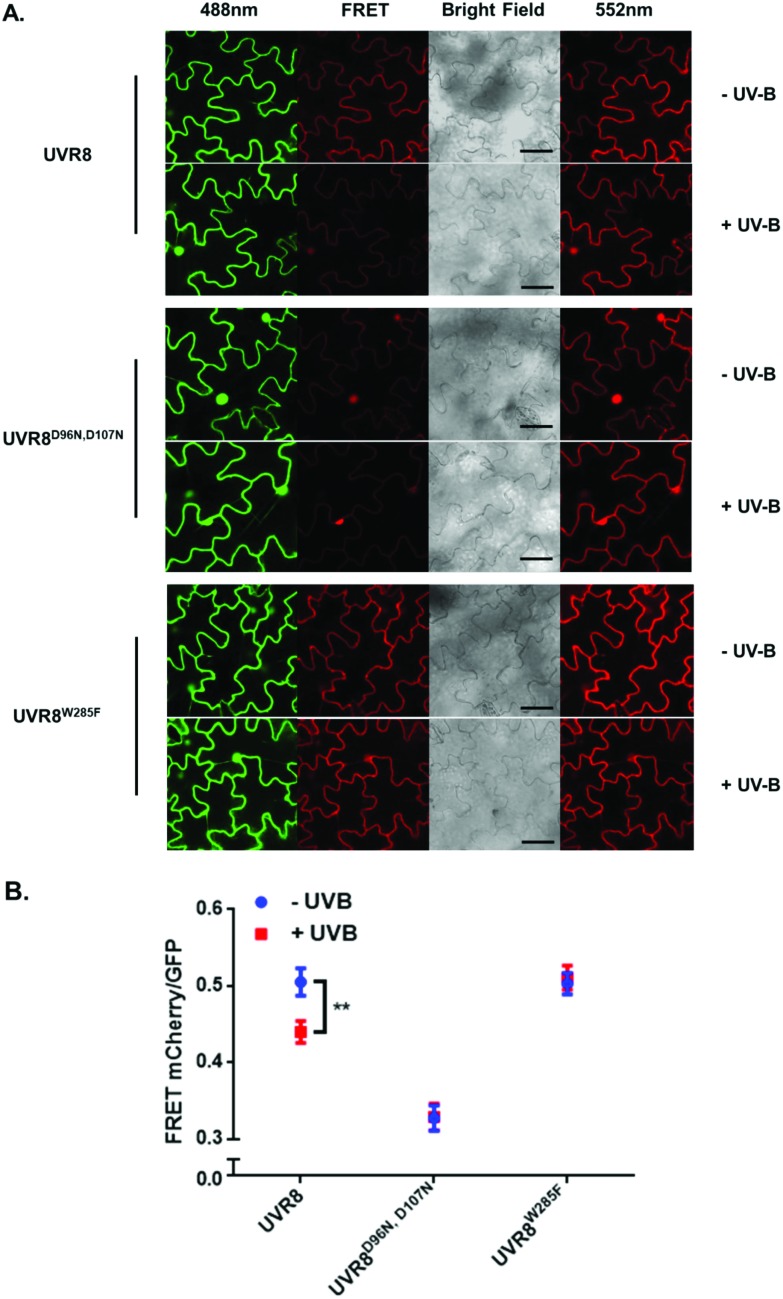
FRET results with the pFRET-2in1-DEST vector. A. Images were captured from tobacco leaves transiently expressing mCherry-UVR8-mCherry/GFP-UVR8-GFP, mCherry-UVR8^D96N,D107N^-mCherry/GFP-UVR8^D96N,D107N^-GFP and mCherry-UVR8^W285F^-mCherry/GFP-UVR8^W285F^-GFP from the pFRET-2in1-DEST vector (fusions abbreviated in the figure panels to UVR8, UVR8^D96N,D107N^ and UVR8^W285F^). Plants were exposed (+UV-B) or not (–UV-B) to 3 μmol m^–2^ s^–1^ broadband UV-B for 1 hour before taking images. Images from left to right are GFP fluorescence signal excited with 488 nm light, mCherry fluorescence signal excited with 488 nm light (FRET), bright field and mCherry fluorescence signal excited with 552 nm light. Bar = 50 μm. B. Means ± S.E. of FRET ratios from 5 independent experiments of constructs in A. For each experiment, data were collected from 10 images and 20 regions of each image were selected at random to calculate the mean fluorescence intensity. FRET efficiency was calculated as the mean fluorescence intensity ratio [mCherry (488)/GFP (488)]. **Indicates significance at *P* < 0.01.

The 2in1 constructs with UVR8, UVR8^D96N,D107N^ and UVR8^W285F^ were transiently expressed in tobacco and checked for protein expression and dimer/monomer status by immunodetection with the appropriate antibodies (Fig. S2†). FRET experiments were performed as described previously and the fluorescence images are shown in Fig. 6A. Quantification of the FRET efficiencies (Fig. 6B) shows that all values are lower than those obtained with the pFRET-NcCg-DEST vector (Fig. 2), possibly because of the loss of intramolecular FRET signals. However, the overall trend for all UVR8 variants is similar to that seen with the pFRET-NcCg-DEST vector. UVR8 and UVR8^W285F^ have a relatively strong FRET signal in the absence of UV-B, whereas UVR8^D96N,D107N^ shows a very weak FRET signal. Wild-type UVR8 shows a FRET signal after UV-B exposure, most likely because of incomplete monomerization and the reversion of some monomers to dimers. Monomeric UVR8^D96N,D107N^ still has a detectable FRET signal, possibly because of weak transient interactions between monomers *in vivo*, which were observed in a previous study.^22^

**Fig. 7:**
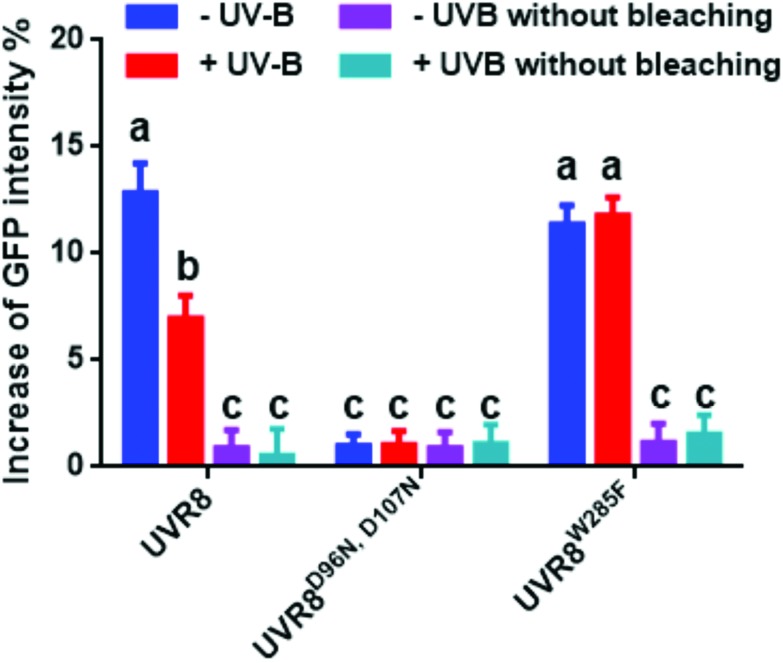
Photobleaching results with the pFRET-2in1-DEST vector. Analysis of GFP fluorescence intensity before and after photobleaching of mCherry-UVR8-mCherry/GFP-UVR8-GFP, mCherry-UVR8^D96N,D107N^-mCherry/GFP-UVR8^D96N,D107N^-GFP and mCherry-UVR8^W285F^-mCherry/GFP-UVR8^W285F^-GFP expressed from the pFRET-2in1-DEST vector (fusions abbreviated in the figure to UVR8, UVR8^D96N,D107N^ and UVR8^W285F^). Plants were exposed (+UV-B) or not (–UV-B) to 3 μmol m^–2^ s^–1^ broadband UV-B for 1 hour before being used for experiments. Primary data (shown in Fig. S4†) were obtained as described in Fig. 3. The percentage increase in GFP fluorescence intensity after photobleaching was calculated by [(*I*_Pb_ – *I*_Pre_)/*I*_Pre_] × 100, where *I*_Pre_ and *I*_Pb_ indicate GFP intensities in the bleaching area before and after photobleaching, respectively. Means with S.E. are shown (*n* > 5). Significance at *P* < 0.01 is indicated by letters.

The photobleaching method was also carried out with these constructs and the primary data are shown in Fig. S4.† The percentage increase in GFP fluorescence after photobleaching was calculated as described previously and results are shown in Fig. 7. The overall trend in the results is similar to that for the FRET efficiency (Fig. 6); UVR8 monomer has a reduced signal relative to the dimer, and for UVR8^D96N,D107N^ there is no significant increase in GFP intensity after photobleaching (Fig. 7), suggesting that essentially no FRET happens for this constitutive monomer mutant.

## Discussion

This study has developed and evaluated FRET-based methods to assess the dimer/monomer status and conformation of UVR8 proteins *in vivo*. The method will be valuable not only for monitoring UVR8 dimer/monomer status in relation to UV-B signaling in the wild-type, but also for the characterization of mutant UVR8 proteins, as demonstrated by the examination of two mutants with distinct molecular phenotypes. Moreover, since the FRET method is used in conjunction with transient expression in tobacco, rapid mutant characterization is possible.

To measure the FRET efficiency, two different approaches were used. The first involves calculation of the fluorescence intensity ratio of acceptor to donor (Fig. 2 and 6). For this method, there are several potential sources of error. First of all, the protein expression level can have a large impact on the results. Overexpression causes the signal to be too strong, and low expression may cause false negative results. Second, because of the spectral overlap of the donor and acceptor, the spectral bleed-through of exciting light may cause a false positive.^24^,^25^ This could explain why UVR8^D96N,D107N^ expressed from the pFRET-2in1-DEST vector still has some FRET signals (Fig. 6). Moreover, when GFP is activated, it excites mCherry almost at the same time, so the GFP fluorescence intensity obtained here is actually the signal after FRET, suggesting that the actual GFP intensity should be higher than the observed value and the ratio of mCherry to GFP should be lower. The second approach employed was acceptor photobleaching, which measures the energy loss of the donor during FRET and therefore avoids the complications associated with measuring the ratio of signals. On the other hand, a potential disadvantage of this method is that it may cause a false negative if the intensity change of the donor is too low to be detected. In this study, both methods were shown to be feasible and to produce similar results. However, the photobleaching method may be more accurate for the reasons stated above.

The two different vectors used in this study enable different molecular properties to be characterized. The pFRET-NcCg-DEST vector is designed to test the conformational change of a single molecular protein and is therefore ideal for the monomeric form of UVR8. However, UVR8 exists in a dimer/monomer photo-equilibrium in light-grown plants and unfortunately the UVR8 dimer cannot be completely removed *in vivo* because monomers continually re-associate to form dimers,^9^ which complicates interpretation. For the UVR8 dimer, as discussed above, there are two different sources of the FRET signal, the intermolecular signal between two monomers and the intra-monomer signal. It is difficult to calculate the relative contribution of these components to the FRET signal, but use of the pFRET-2in1-DEST vector^23^ can overcome this problem because only intermolecular FRET is possible. A major advantage of this vector is that it can express two different gene fusions in a 1 : 1 ratio, ensuring both proteins are expressed at very similar levels. In this study, it was used to assay FRET signals between two differently labeled UVR8 monomers. However, the dimer/monomer photo-equilibrium still probably affects the result. The differently labeled monomers will randomly combine to form three kinds of dimers, but only dimers of GFP-UVR8-GFP with mCherry-UVR8-mCherry will generate FRET. Dimers with the same tags, GFP-UVR8-GFP with GFP-UVR8-GFP or mCherry-UVR8-mCherry with mCherry-UVR8-mCherry, do not contribute to the FRET signal, but their fluorescence will interfere with the FRET efficiency calculation.

The results obtained for UVR8 and the two mutants are largely consistent with previous reports but also raise some interesting points. UV-B induced monomerization of wild-type UVR8 strongly reduces the FRET signal in all of the experiments shown here. It is interesting that the results with the pFRET-NcCg-DEST and pFRET-2in1-DEST vectors are similar, because this suggests that there is relatively little, if any, change in the intramolecular FRET signal, and hence little UV-B induced change in UVR8 conformation. Several previous studies using a number of techniques have provided evidence for conformational change of UVR8 associated with UV-B photoreception.^5^,^10^–^12^ In addition, a recent report shows that the monomer can adopt multiple conformations, including partial unraveling of the β-propeller.^13^ Production of the most extended conformation may be required to facilitate interaction with other proteins, in particular at the C-terminal region, to initiate signaling. However, the above studies of conformational changes were undertaken with purified protein, whereas the present experiments were with UVR8 expressed *in vivo*, the major difference being that UVR8 is in a cellular environment and can bind to proteins, such as the RUPs, that may stabilize the structure. An additional point to consider is that detection of a change in the intramolecular FRET signal depends on a change in distance between the N- and C-termini of the protein, and it is quite possible for conformational changes to occur without much change in their relative positions. It will therefore be valuable to undertake further studies of UVR8 conformational change *in vivo* using additional approaches.

The FRET results for UVR8^W285F^ are consistent with it being a constitutive dimer that is unresponsive to UV-B treatment, as reported previously both for the purified protein and *in vivo*.^3^–^5^,^21^,^26^ UVR8^D96N,D107N^ is a constitutively monomeric mutant,^22^ and is therefore not expected to show an intermolecular FRET signal. The very low signal seen using photobleaching with the pFRET-2in1-DEST vector is likely to be due to either transient, weak interaction between monomers^22^ and/or a false positive caused by spectral bleed-through of the exciting light, as mentioned above. Since intermolecular FRET is very low, the pFRET-NcCg-DEST vector is suitable to investigate any conformational change of UVR8^D96N,D107N^. The results (Fig. 2C and 4) show that UV-B exposure does not change the FRET efficiency, indicating that there is no conformational change, or at least no change that significantly alters the relative positions of the N- and C-termini. UVR8^D96N,D107N^ is functional *in vivo* in initiating responses to UV-B. However, in contrast to wild-type UVR8, UVR8^D96N,D107N^ can constitutively interact with COP1, although binding does increase after UV-B exposure. Hence, the mutation may produce a protein conformation that is normally only produced in wild-type UVR8 by UV-B photoreception. One possibility is that the UVR8^D96N,D107N^ monomer adopts one of the extended forms described by Camacho *et al.* (2018),^13^ enabling it to interact with COP1, and that UV-B photoreception further activates the protein to generate the signaling-active state. Research to date therefore suggests that UV-B plays at least two roles in UVR8 signaling: inducing the monomerization of UVR8 homodimer, which is necessary for UVR8 to interact with COP1, and activating UVR8 monomer to initiate signaling.

## Conflicts of interest

There are no conflicts of interest to declare.

## Supplementary Material

Supplementary information

Supplementary information
