# Supplementary material for: A FRET method for investigating dimer/monomer status and conformation of the UVR8 photoreceptor
Source: Photochem Photobiol Sci. 2018 Dec 4;18(2):367–74. doi: 10.1039/c8pp00489g (PMC6374739; doi:10.1039/c8pp00489g)
Supplement: Supplementary file 1 [file PP-018-C8PP00489G-s001.pdf]

Electronic Supplementary Material (ESI) for Photochemical & Photobiological Sciences.
This journal is © The Royal Society of Chemistry and Owner Societies 2018

UVR8 F-attB1	GGGGACAAGTTTGTACAAAAAAGCAGGCTTAATGGCGGAGGATATGGCTGC
UVR8 F-attB3	GGGGACAACTTTGTATAATAAAGTTGTAATGGCGGAGGATATGGCTGC
UVR8 R-attB2 (with stop)	GGGGACCACTTTGTACAAGAAAGCTGGGTTCAAATTCGTACACGCTTGAC
UVR8 R-attB2 (without stop)	GGGGACCACTTTGTACAAGAAAGCTGGGTGAATTCGTACACGCTTG
GFP R-attB2	GGGGACCACTTTGTACAAGAAAGCTGGGTGTCACTTGTACAGCTCGTCCATGCCG
mCherry R-attB4	GGGGACAACTTTGTATAGAAAAGTTGGGTGTCACTTGTACAGCTCGTCCATGCCG
Overlap F	GTACGAATTATGGTGAGCAAG
Overlap R	GCTCACCATAATTCGTACACG

Table S1 Primers used in making constructs.
