# Supplementary material for: A FRET method for investigating dimer/monomer status and conformation of the UVR8 photoreceptor
Source: Photochem Photobiol Sci. 2018 Dec 4;18(2):367–74. doi: 10.1039/c8pp00489g (PMC6374739; doi:10.1039/c8pp00489g)
Supplement: Supplementary file 2 [file PP-018-C8PP00489G-s002.pdf]

This journal is © The Royal Society of Chemistry and Owner Societies 2018

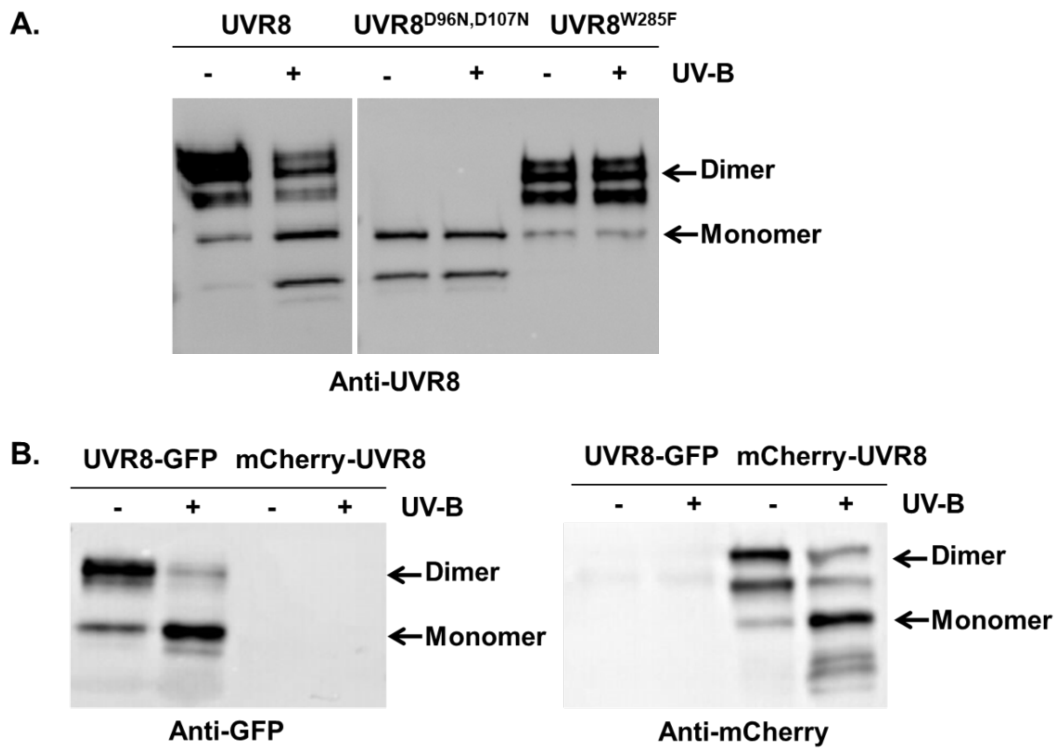

Figure S1. Immunoblots of tobacco leaf samples used in Figures 2 and 4.

A. Protein immunoblots of tobacco leaf samples transiently expressing mCherry-UVR8-GFP, mCherry-UVR8^{D96N,D107N}-GFP and mCherry-UVR8^{W285F}-GFP from the pFRET-NcCg-DEST vector (fusions abbreviated in the Figure to UVR8, UVR8^{D96N,D107N} and UVR8^{W285F}), probed with anti-UVR8 C-terminus antibody described by Kaiserli and Jenkins (2007; *Plant Cell* 19, 2662-73). Leaf protein extraction and immunodetection were carried out as described previously (Kaiserli and Jenkins, 2007). Resolution of UVR8 dimer and monomer bands was achieved using SDS polyacrylamide gel electrophoresis with non-boiled samples (Rizzini *et al.* 2011; *Science* 332, 103-6), using the method detailed by Heilmann and Jenkins (2013; *Plant Physiol.* 161, 547-55). Plants were exposed (+ UV-B) or not (- UV-B) to 3 μmol m^{-2} s^{-1} broadband UV-B for 1 hour. **B.** Protein immunoblots of tobacco leaf samples transiently expressing UVR8-GFP and mCherry-UVR8. Left, probed with anti-GFP antibody (Takara Clontech); Right, probed with anti-mCherry antibody (Abcam). Plants were exposed or not to UV-B as in A.

Bands with mobility between dimer and monomer likely represent intermediate conformations of UVR8 produced during dimer-monomer conversion, which have been

observed in previous studies. Bands running below the monomer are likely proteins lacking the C-terminal GFP tag, based on antibody detection. The loss of one tag will not influence FRET, which requires the presence of both GFP and mCherry.

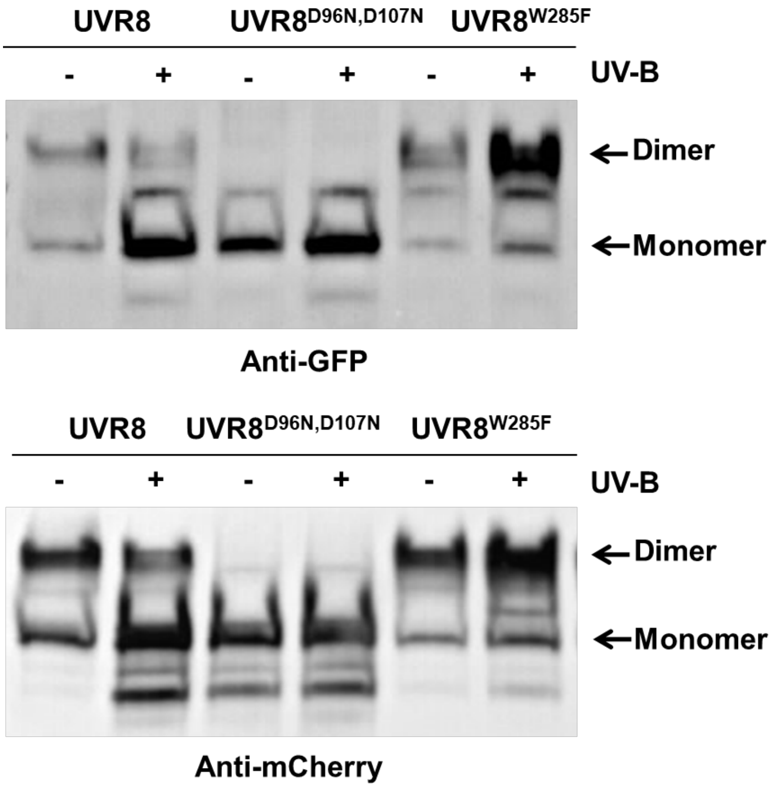

Figure S2. Immunoblots of tobacco leaf samples used in Figure 6 and Figure 7.

Protein immunoblots of tobacco leaf samples transiently expressing mCherry-UVR8-mCherry/GFP-UVR8-GFP, mCherry-UVR8^{D96N,D107N}-mCherry/GFP-UVR8^{D96N,D107N}-GFP and mCherry-UVR8^{W285F}-mCherry/GFP-UVR8^{W285F}-GFP from the pFRET-2in1-DEST vector (fusions abbreviated in the Figure panels to UVR8, UVR8^{D96N,D107N} and UVR8^{W285F}). Plants were exposed (+ UV-B) or not (- UV-B) to 3 μmol m^{-2} s^{-1} broadband UV-B for 1 hour. Immunodetection was performed as described in Figure S1. Above, probed with anti-GFP antibody (Takara Clontech); Below, probed with anti-mCherry antibody (Abcam). Bands running between dimer and monomer and below the monomer are explained in Figure S1.

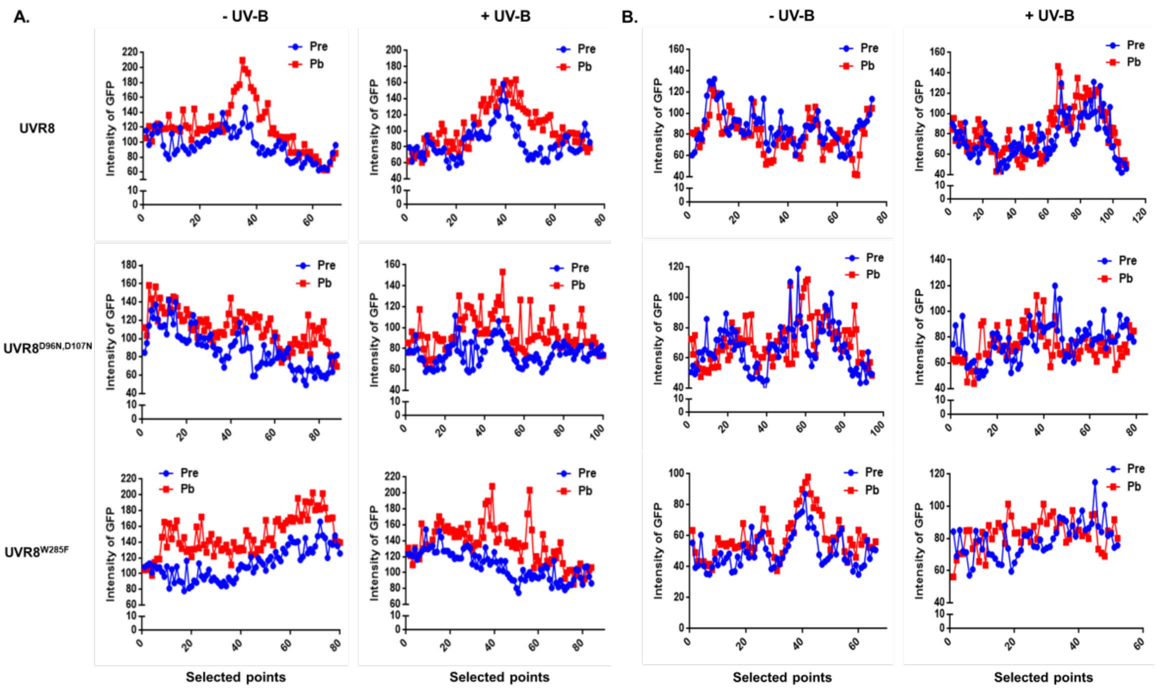

Figure S3. Photobleaching results with the pFRET-NcCg-DEST vector.

A. Analysis of GFP fluorescence intensity of mCherry-UVR8-GFP, mCherry-UVR8^{D96N,D107N}-GFP and mCherry-UVR8^{W285F}-GFP expressed from the pFRET-NcCg-DEST vector (fusions abbreviated in the Figure to UVR8, UVR8^{D96N,D107N} and UVR8^{W285F}), before and after photobleaching. Images were obtained as described in Figure 3. Plants were exposed (+ UV-B) or not (- UV-B) to 3 μmol m^{-2} s^{-1} broadband UV-B for 1 hour before being used for experiments. **B.** The negative control for results in A.

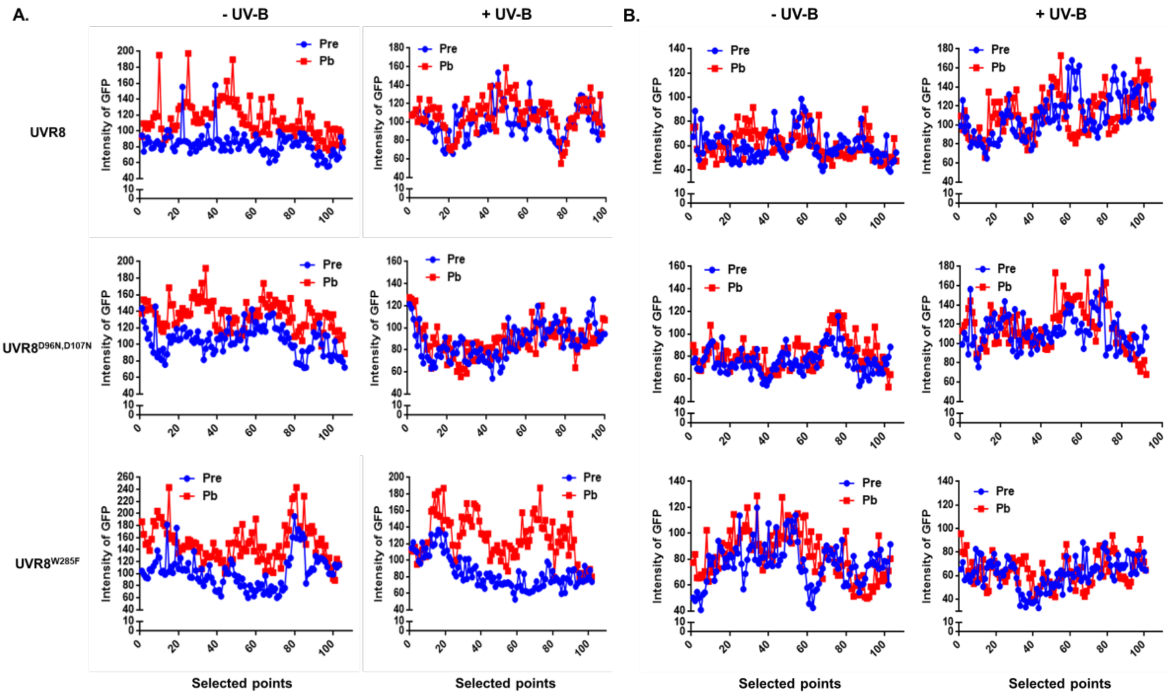

Figure S4. Photobleaching results with the pFRET-2in1-DEST vector.

A. Analysis of GFP fluorescence intensity of mCherry-UVR8-mCherry/GFP-UVR8-GFP, mCherry-UVR8^{D96N,D107N}-mCherry/GFP-UVR8^{D96N,D107N}-GFP and mCherry-UVR8^{W285F}-mCherry/GFP-UVR8^{W285F}-GFP from the pFRET-2in1-DEST vector (fusions abbreviated in the Figure panels to UVR8, UVR8^{D96N,D107N} and UVR8^{W285F}), before and after photobleaching. Images were obtained as described in Figure 3. Plants were exposed (+ UV-B) or not (- UV-B) to 3 μmol m^{-2} s^{-1} broadband UV-B for 1 hour before being used for experiments. **B.** The negative control for results in A.
